# Supplementary material for: Learning curve and surgical outcome of robotic assisted colorectal surgery with ERAS program
Source: Sci Rep. 2022 Nov 29;12:20566. doi: 10.1038/s41598-022-24665-w (PMC9709162; doi:10.1038/s41598-022-24665-w)
Supplement: Supplementary file 2 — Supplementary Information 2. [file 41598_2022_24665_MOESM2_ESM.zip › RAL Raw data-Ñ[▒K/122/HS_ñw╝╨░O▒Kñσ.pdf]

正式摘要(電子簽章：00000229120898)<br>W52 / W52 ☐ 英文標題(注意:無英文姓名-請補病人基本檔!)	☐ 【讀取】憑證	

臺中榮民總醫院

出院病歷摘要

轉入號 : 轉出號 :

[redacted]

**(8)**流水編號	
**(9)**入院日期	110/10/29 (2021) 直腸外科 W52 - 162
**(10)**轉科日期	
**(11)**出院日期	110/11/02 (2021) 住院天數計 4 日

診斷

- 入院 : 1. S-colon cancer, FAV 20cm
  2. HTN
- 出院 : 1. S-colon cancer, FAV 20cm, s/p Robotic assist AR on 2021/10/30, T4N0M0
  2. HTN

主訴

- progressed bloody stool recently

病史

- This 63 years old male patient has past history of hypertension. He complained smaller caliber stool with blood-tinged stool for about 6 months. Bloody stool progressed in recent months. He came to LMD for help. Sigmoidoscope showed tumor at sigmoid colon, 20 cm FAV that malignancy was suspected. He was referred to our hospital. No obvious distal metastasis was found in abdominal CT. Thus, he was admitted for surgical treatment.

體檢發現

-
  - General appearance :
    - easy looking , conscious clear , without cardiopulmonary distress
    - Vital signs自動帶入date 體溫(BT) 脈搏(P) 呼吸(R) 血壓(BP) 身高(BH) 體重(BW) BMI
      20211029 36.4℃ 72次/分 16次/分 120/81mmHg 168.5cm 73.6kg 25.9
  - HEENT :
    - Eyes: no anemic conjunctiva, no icteric sclera, normal EOM
    - ENT: no otorrhea, no rhinorrhea, no erythematous mucosa, no injected tonsil, no deformity
  - Neck: supple, freely movable, trachea midline,no deformity, no engorged jugular vein , no palpable thyroid or lymph node enlargement
  - Chest:
    - Inspection: Grossly normal, symmetric and fully expansion, no accessory muscle used, no operation scar, no spider angiomata, no gynecomastia
    - Palpation: no palpable mass or nodule, no subcutaneous emphysema, no tenderness
    - Ausculation: clear breathing sound
  - Heart:
    - Percussion: normal heart size
    - Ausculation: regular heart beat, normal S1, S2, no gallop, no murmur
  - Abdomen:
    - Inspection: in
      - flat contour, symetrically, no scar, no stria, no dilated veins, no rashes
    - Ausculation: normoactive bowel sound, no abdominal bruits
    - palpation: soft, no muscle guarding or rigidity, no local tenderness, no rebounding pain, no palpable mass, no palpable pulsation

-
    - Percussion: no shifting dullness, no hepatomegaly, no splenomagaly
Peripheral pulsation :	CA	BA	RA	FA	PA	PTA	DPA
Right side :	++	++	++	++	++	++	++/+++
Left side :	++	++	++	++	++	++	++/+++
- Genito-anus: no tenderness or palpable mass lesion or rectal shelf, normal external genitalia, normal anal tone, brown stool

CRS CONDITION

- CEA: 1.61 ng/ml
-
    - Lung

手術日期及方法

- 手術日期及方法20211030 Robot Assisted Colectomy(L)

住院治療經過

- After admission, he received Robiotic assist AR on 2021/10/30. Foley was removed on 10/31 and he start try bland II diet since 11/01. Due to relative stable condition, he was discharged o n2021/11/02.

合併症

一般檢查

- 累積報告 :

DATE	TIME	ALB	TP	BIL.T	BIL.D	ALKP	AST	ALT	LDH	AMY	LIPASE	NH3	Ferritin	UA
1101030	1447	3.9		0.59	0.16		18	12						
1101102	0844	3.3		0.98	0.25		13	6						

DATE	TIME	WBC	RBC	HGB	HCT	MCV	PLT	BLST	NEUT	BAND	SEG	LYM	MON	EOS	BAS
1101030	1443	13640		12.4			224		95.4			3.2	1.2	0.0	0.2
1101102	0857	11920	4.42	11.4	36.9	83.5	226		82.4			9.9	4.9	2.4	0.4

DATE	TIME	NA	K	CL	BUN	CREAT	eGFR	CA	Mg	P	CRP	ESR	PCT	D-Dimer	LACTATE
1101029	1731				21	1.43									
1101030	1447	139	3.2		15	1.19									
1101102	0844	141	3.5		10	1.22	63.77	8.0							

特殊檢查

病理報告

其它

出院時情況

- 3.改門診治療

出院指示

藥名	劑量	劑量單位	用法	頻次	數量	數量單位	現況	天數	單位
(口)Takepron OD tab 30mg	1	TAB	PO	QDAC	7	TAB	TH	7	天
Ultracet tab	1	TAB	PO	Q6H	28	TAB	TH	7	天
BethanechoL tab 25mg-JS	1	TAB	PO	TIDAC	21	TAB	TH	7	天

出院計劃

- 門診日期
    - date on 2021/11/09

主治醫師 2062F 蔣鋒帆 / 住院醫師 2339C 蔡濬樟

正式摘要(電子簽章：000000229120898)

W52 / W52

doctorRank:B
